# Composite Anxiety-depression among Medical Undergraduates during COVID-19 Pandemic in a Tertiary Care Hospital: A Descriptive Cross-sectional Study

**DOI:** 10.31729/jnma.6947

**Published:** 2021-09-30

**Authors:** Pratikshya Chalise, Avilasha Singh, Era Rawal, Pravash Budhathoki, Satyasuna Kafle, Pallawi Jyotsana, Riju Kafle

**Affiliations:** 1 Department of Psychiatry, Kathmandu Medical College, Sinamangal, Kathmandu, Nepal; 2 Kathmandu Medical College, Sinamangal, Kathmandu, Nepal; 3 Norvic International Hospital, Thapathali, Kathmandu, Nepal; 4 Department of Emergency Medicine, Dr. Iwamura Memorial Hospital, Salaghari, Bhaktapur 44800; 5 Bhaktapur Hospital, Ministry of Social Development Health Directorate, Province-3, Bhaktapur Nepal; 6 Nepal Heart Foundation, Nepal Research Associate, Kathmandu, Nepal

**Keywords:** *anxiety*, *COVID-19*, *depression*, *medical students*

## Abstract

**Introduction:**

Fear and anxiety is a natural response during crisis. From constant worry of getting infected, death of loved ones, transitioning of lifestyle to loss of social connection; there can be several psychological triggers. The effect on mental health on the general population could be greater than those affected by the infection itself during the pandemic. Among medical students, who already have several other psychological afflictions, these triggers might be detrimental. Hence, this study aims to find out the prevalence of composite anxiety-depression among medical undergraduates in a tertiary care hospital.

**Methods:**

A descriptive cross-sectional study was conducted among medical undergraduates in a tertiary care hospital from 20th December 2020 to 5th January 2021. Ethical approval was taken from the Institutional Review Committee. The sample size was calculated and convenient sampling was done. The data were entered in International Business Machines Statistical Package for Social Sciences version 20.0. Point estimate at 95% Confidence Interval was calculated along with frequency and proportion for binary data.

**Results:**

Out of 315 participants on the Patient Health Questionnaire-Anxiety and Depression Scale, severe composite anxiety-depression was seen in 10 (3.17%) at 95% Confidence Interval (1.23-5.1) participants, moderate in 35 (11.11%) at 95% Confidence Interval (7.63-14.58) and mild in 98 (31.11%) at 95% Confidence Interval (25.99-36.22). And 172 (54.6%) at 95% Confidence Interval (49.1-60) were normal.

**Conclusions:**

The rapid rise of apprehension among people amidst infectious outbreaks can ensue and medical students are no exception. So, at this time of crisis, there is a need to protect their mental health and it should be emphasized and endorsed.

## INTRODUCTION

With the surge in COVID-19 pandemic, in Nepal, there have been 6,58,778 total reported cases, among which 26,261 are actively infected and 9,412 confirmed deaths due to COVID-19,^1^ and the cases are still on the rise. In a crisis like this fear and anxiety is a natural response.^2^

Since the pandemic started, apprehension of contracting infection, change in lifestyle associated with lockdown, mass quarantine, death of loved ones has created an insurmountable psychological pressure among general population, which may lead to various psychological problems, such as anxiety, depression, and insomnia.^3^ Psychological affliction among medical students tends to be greater than their non-medical contemporaries in itself.^4^ Add on to that, the damaging effect on mental health among them during pandemic and subsequent lockdown could have been escalated. Hence it is important to find out the prevalence of these problems so that early and appropriate interventions can be done.

This study aims to find out the prevalence of composite anxiety-depression among medical undergraduates in a tertiary care hospital.

## METHODS

A descriptive cross-sectional study was conducted in Kathmandu Medical College and Teaching Hospital (KMCTH), Sinamangal, Kathmandu, Nepal. After taking the ethical clearance from the Institutional Review Committee of KMCTH with reference number 2611202001, data were collected from medical undergraduates (MBBS, BDS, and B.Sc. Nursing Students) from first to final year studying at KMCTH, Kathmandu from 20^th^ Dec 2020 to 5^th^ Jan 2020. The sample size was calculated using the formula,

n = Z^2^ × p × q / e^2^

  = (1.96)^2^ × 0.5 × (1-0.5) / 0.05^2^

  = 0.9604 / 0.0025

  = 384.16

where,

n = required sample size
p = prevalence 50 % taken
q = 1-p
e = margin of error, 5%
Z = 1.96 at 95% Confidence Interval

Taking the finite population i.e., total medical undergraduates of Kathmandu Medical College (N)= 1000

Adjusted sample size = n / {1+(n-1) / N}

    = 384.16 / {1+(384.16-1) / 1000}

    = 277.74

    ≈278

Therefore, the calculated sample size was 278. Adding the non-response rate of 10%, the sample size was 305.8 i.e., 306. A total of 315 participants who gave their consent were included in the study.

Convenient sampling was done and a self-administered online questionnaire containing; demographic information, self-framed semi-structured questionnaire on impact of COVID-19, Generalised Anxiety Disorder Assessment (GAD-7), and Patient Health Questionnaire (PHQ-9) were circulated to the participants via Google Forms.

GAD-7 is one of the customarily used diagnostic selfreport scales for screening, diagnosis, and severity assessment of anxiety disorder. It has good reliability and procedural validity.^5^ The PHQ-9 makes the criteria-based diagnosis of depressive disorders. It is a reliable and valid measure of depression severity.^6^

For GAD-7 scoring, cut off point 5, 10 and 15 were taken. Where score 0 to 4 were categorized as no anxiety, 5 to 9 as mild anxiety, 10 to 14 as moderate, 15 and above as severe anxiety. Similarly, for PHQ-9 scoring, 0-4 were categorized as none, 5-9 mild, 10-14 moderate, 15-19 as moderately severe, 20-27 as severe depression. Then as per the Patient Health Questionnaire-Anxiety and Depression Scale (PHQ-ADS) the sum of GAD-7 and PHQ-9 was calculated and categorized into normal, mild, moderate and severe category using the cutoff point 10, 20 and 30.^7^ To minimize the information bias the participants were assured about the confidentiality of their data and validated.

The data was entered in International Business Machines Statistical Package for Social Sciences (IBM SPSS) version 20.0. Point estimate at 95% Confidence Interval was calculated along with frequency and proportion for binary data.

## RESULTS

Out of 315 students included in the study on the PHQ-ADS scale, 143 (45.39%) students had different grades of composite anxiety-depression. Ten (3.17%) at 95% Confidence Interval (1.23-5.1) were found to be on the severe category, 35 (11.11%) at 95% CI (7.63-14.58) fell on the moderate composite anxiety-depression category and 98 (31.11%) at 95% CI (25.99-36.22) were on the mild category. No composite anxiety-depression was seen in 172 (54.6%) at 95% CI (49.1-60).

Similarly, discerning only anxiety on GAD-7 scoring the prevalence of severe anxiety was found to be 10 (3.17%). Also, moderate anxiety was seen in 29 (9.2%) and mild anxiety was seen in 115 (36.5%) students. Finally, anxiety was not seen in 161 (51%) students. Again, on discerning only depression on PHQ-9 scoring the prevalence of severe depression was found to be 7 (2.22%), moderately severe depression was seen in 15 (4.76%) students, moderate depression in 43 (13.65%), mild depression was seen in 76 (24.12%) students, and no depression in 174 (55.23%) students.

The demographic profile of the participants was as shown below (Table 1).

**Table 1:** Demographic profile of the participants.

Demographic profiles		n (%)
Age	18-19	32 (10.15)
	20-21	173 (54.92)
	22-23	82 (26)
	24-25	28 (8.88)
Sex	Male	109 (34.6)
	Female	206 (65.4)
Professional degree	BDS	68 (21.6)
	MBBS	164 (52.1)
	Nursing	83 (26.3)
Year in medical journey	First-year	66 (21)
	Second-year	124 (39.4)
	Third-year	90 (28.6)
	Fourth-year	35 (11.1)

On sex-wise subgroupanalysis ofthe PHQ-ADSScore, out of 109 males, 23 (21%) were seen to be on the mild composite anxiety-depression category, 11 (10.09%) were on moderate category and 5 (4.58%) were on severe category. Seventy (64.22%) were without composite anxiety-depression. Similarly, out of 206 females, 102 (49.51%) were normal, 75 (36.4%) were in the mild category, 24 (11.65%) were in the moderate and 5 (2.42%) were in the severe category.

Likewise, out of 66 first-year students 2 (3.03%) fell on the severe composite anxiety-depression category. While among 35 final year students 6 (17.14%) were in the same category.

Ten (3.2%) students had pre-existing psychiatric illnesses. Among them 7 (70%) out of 10 had General Anxiety Disorder (GAD), one (10%) had bipolar disorder and one (10%) response was present for post-traumatic stress disorder (PTSD). One (10%) student reported having GAD, PTSD, and obsessive-compulsive disorder (OCD). Four (40%) students out of 10 had been taking medication for their conditions. Among them, 7 out of 10 (70%) responded that their symptoms had worsened during this period.

Thirteen (4.1%) participants reported that they were adapting very easily with day-to-day life with the emergence of COVID-19 pandemic, 81 (25.7%) said somewhat easily, 75 (23.8%) said somewhat difficulty, whereas 5 (1.6%) were adapting very difficulty. One hundred and forty-one (44.8%) were in between. When asked how much had COVID-19 affected life overall, 13 (4.1%) said they were affected a great deal, 88 (27.9%) were affected a lot, 162 (51.4%) a moderate amount, 45 (14.3%) a little whereas 7 (2.2%) were not affected at all.

Regarding the effect on lifestyle, 25 (7.9%) were always worried while going to buy the groceries and 27 (8.6%) were never, remaining were in between.

Seventy-one (22.5%) participants were always exposed to news and information regarding COVID-19 on social media whereas 4 (1.3%) were never exposed, others were in between. 7 (2.2%) said that they distrusted all the information on COVID-19, 256 (81.3%) distrusted some information whereas 52 (16.5%) did not distrust any information.

**Figure 1:**
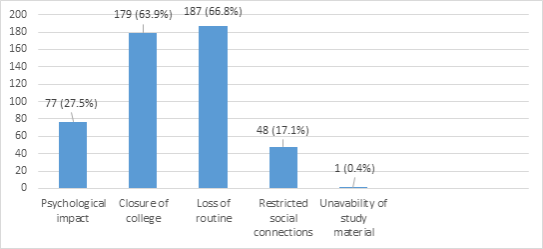
Reasons for effect in the studies.

When asked about the effect in studies 282 (89.5%) reported that their studies were affected. Then when asked about the reason for effect in their studies they reported as follows (participants were allowed to choose multiple options) (Figure 1).

Eighty-three (26.3 %) were always worried about the effect of the COVID-19 pandemic on their academic delay, 110 (34.9%) were usually worried, 105 (33.3%) were sometimes worried, 13 (4.1%) were rarely worried and 4 (1.3%) were never worried. One hundred and twenty-three (39%) said that their future plans changed due to the outbreak of the COVID-19 pandemic.

**Figure 2:**
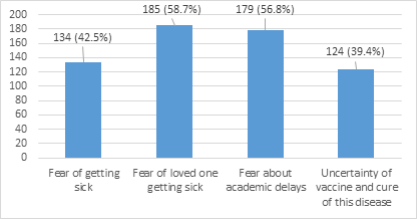
Reasons for fear during COVID-19.

Twenty-six (8.3%) participants reported that they always felt threatened on thinking about COVID-19 whereas 13 (4.1%) did not feel threatened at all. One hundred forty three (45.4%) reported that they sometimes felt threatened. Then they were asked about what they feared the most (Figure 2).

Thirteen (4.1%) respondents did not fear anything. Compared to the beginning of the pandemic, the level of concern had increased in 47 (14.9%), decreased in 229 (72.7%) whereas unchanged in 39 (12.4%).

## DISCUSSION

In a study conducted to assess the prevalence of depression and anxiety among the Nepali population during the COVID-19 lockdown, the overall prevalence rates of depression, anxiety and depression, and anxiety co-morbidity were found to be 34.1%, 31.2%, and 23.2% respectively which was higher compared to our study.^3^ Since the study was done among the general population so lack of detail knowledge regarding the disease, its progression and prognosis may have contributed to higher mental health impact and apprehension as compared to our study where only medical undergraduates were taken who have clearer understanding of the disease and know what to expect, which may have decreased their stress level.

Similarly in another study done in Nepal at the community level, 66.1% of the study population were worried about their families being infected and 60.4% continued updating with COVID-19 related news and information whereas in our study it was 58.7% and 22.5% respectively.^8^ The study showed higher psychological impact among females which was a similar finding in our study as well.

Also in another study conducted among individuals attending a tertiary care hospital in Kathmandu, 66.7% of the sample experienced stress which was higher than seen in our study.^9^ This might have been because the study population included patients having comorbidities visiting the hospital during lockdown, which might have amplified their stress level. Similarly, the study showed no significant difference between stress among male and female^9^ where as in our study female were comparatively more in mild composite anxiety-depression category than males. In the same study out of the participants with pre-existing conditions, 31.3% reported to have exacerbations of the psychological symptoms, which was lower than in our study, which might have been due to the larger study sample pool in the former.

A study done among Iranian medical students showed severe anxiety among 4.6% and severe depression among 2.8%, which was somewhat similar to our study. Similarly in another study done among medical students in China, 0.9% of the respondents were experiencing severe anxiety, 2.7% moderate anxiety, and 21.3% mild anxiety which was less than in our study.^11^ Also, our result was similar to a study conducted among medical students in the United States which showed 30.6% anxiety.^12^ In this study by Halperin et al, anxiety and depression was seen more among first year and second year medical students (pre-clinical) compared to third year and final year.^12^ But in our study composite anxiety-depression category was higher among the final year medical students. This might have been due to the different study setting in the western and eastern countries.

In a survey conducted in China during the initial outbreak of COVID-19, 16.5% of respondents reported moderate to severe depressive symptoms; 28.8% reported moderate to severe anxiety symptoms which were relatively high compared to ours which showed 3.17% and 2.22% severe anxiety and depression respectively.^13^ This might have been because the study was conducted quite earlier than ours when the public's apprehension was at its peak and the knowledge regarding COVID-19 was very minimal. Likewise in a study done among the Liaoning Province in China, 52.1% of participants reported that they felt horrified and apprehensive due to the COVID-19 pandemic^14^ whereas in our study 26 (8.3%) participants reported that they always felt threatened on thinking about COVID-19. The differences seen in the study could be because of the difference in study design.

Our study was conducted in only one medical college hence the findings cannot be generalized among the general population. Since a descriptive cross-sectional study was conducted, the prevalence of anxiety and depression could only be quantified but the association to triggers/stressors resulting from the pandemic could not be established. The online survey and convenient sampling method may also create some limitations.

## CONCLUSIONS

The prevalence of composite anxiety-depression among medical undergraduates in our study is in comparison to the similar study done in a similar setting. The rapid rise of apprehension among people amidst infectious outbreaks is an understandably natural phenomenon, and medical students are no exception to this. Mental and physical health are interwoven strands of life. Hence it is of utmost importance to take care of not only physical but also mental health as well. As the concept of "mental health is essential" is on the rise; at this time of crisis, it should be emphasized and endorsed.
